# Effects of Fermentation on the Bioactive, Functional, and Technological Characteristics of Babassu and Brazil Nut Flours

**DOI:** 10.1111/1750-3841.70554

**Published:** 2025-09-11

**Authors:** Letícia Nunes da Cruz, Beatriz Veltre Costa, Caroline Lopes, Cecília Midori Hosoda Henriques, Ruann Janser Soares de Castro

**Affiliations:** ^1^ Department of Food Science and Nutrition, School of Food Engineering Universidade Estadual de Campinas Campinas São Paulo Brazil

**Keywords:** antidiabetic, antioxidant, *Attalea speciosa*, *Bertholletia excelsa*, *Saccharomyces cerevisiae* var. *boulardii*

## Abstract

This study provides a comprehensive evaluation of the composition, bioactivity, and techno‐functional properties of babassu (*Attalea speciosa*) mesocarp and Brazil nut (*Bertholletia excelsa*) flours, highlighting their potential as novel functional ingredients. Fermentation effects of *Saccharomyces cerevisiae* var. *boulardii*‐17 on these properties were also investigated. Yeast cells (10^8^ cells mL^−1^) were inoculated into flour suspensions (100 mg mL^−1^), and samples were collected every 4 h over a 12‐h fermentation period. In babassu flour, fermentation increased protein content and reduced lipid and ash levels, whereas Brazil nut flour showed minimal compositional changes, except for a decrease in carbohydrates. Lysine content increased in both substrates after fermentation. Among fermented samples, the 12‐h fermented babassu flour showed the best results for antioxidant assays, reaching 92.76, 105.07, and 181.77 µmol TE g^−1^ for 2,2′‐azino‐bis(3‐ethylbenzothiazoline‐6‐sulfonic acid) (ABTS), 2,2‐difenil‐1‐picrilhidrazil (DPPH), and ferric reducing antioxidant power (FRAP) methods, respectively. Brazil nut samples also showed improved antioxidant activity after 4 h, with DPPH and FRAP values of 6.48 and 11.77 µmol TE g^−1^, respectively. For babassu, α‐glucosidase inhibition peaked after 8 h of fermentation (40.26%) and remained high after 12 h (35.87%). Fermentation enhanced the emulsifying capacity of Brazil nut flour, exceeding 74% from 4 h onward. Upon scale‐up, improvements in ABTS and DPPH scavenging activity, α‐glucosidase inhibition, and oil holding capacity were detected in babassu, whereas Brazil nut flour showed enhanced gelling properties. The results suggest the potential application of these fermented flours as functional ingredients in the formulation of plant‐based food products.

**Abbreviations**
ABTS: 2,2′‐azino‐bis(3‐ethylbenzothiazoline‐6‐sulfonic acid)
CFU: colony forming unit
CGC: critical gelling concentration
DPPH: 2,2‐difenil‐1‐picrilhidrazil
EC: emulsifying capacity
FBMF: fermented babassu mesocarp flour
FBMS: babassu mesocarp flour fermented in bioreactor
FBNF: fermented Brazil nut flour
FBNS: Brazil nut flour fermented in bioreactor
FRAP: ferric reducing antioxidant power
NFBMF: non‐fermented babassu mesocarp flour
NFBNF: non‐fermented Brazil nut flour
OHC: oil holding capacity
TPTZ: 2,4,6‐tris(2‐pyridyl)‐*S*‐triazine
WHC: water holding capacity
ρNPG: *ρ*‐nitrophenyl α‐d‐glucopyranoside

## 1 Introduction

The global demand for plant‐based ingredients has grown substantially in recent years, driven by increasing consumer interest in health, sustainability, and ethical food choices. According to Rogers et al. (2024), sales of plant‐based foods that directly replace animal products increased by 53% between 2019 and 2021. This rapid growth underscores the need for the research and development sector to provide ingredients that are not only technologically suitable but also capable of delivering health‐promoting components, such as antioxidants, dietary fiber, and probiotics (Noguerol et al. 2021).

Fermentation is a versatile biotechnological method for enhancing the nutritional and functional value of plant‐derived ingredients. It can increase both the content and bioavailability of bioactive compounds while simultaneously enabling the incorporation of probiotic microorganisms into the fermented product (Zhao et al. 2025; Hu et al. 2025). In addition, fermentation modifies macromolecular structures, such as protein and starch, through the action of microbial enzymes, thereby improving key techno‐functional properties (Çabuk et al. 2018). These enhancements are especially relevant for the formulation of plant‐based beverages, which require good solubility, emulsion stability, and textural consistency (Kim et al. 2025; Sharma et al. 2024).

This study explores the use of two underutilized Amazonian plant flours—babassu (*Attalea speciosa*) mesocarp flour and Brazil nut (*Bertholletia excelsa*) flour—as fermentation substrates for *Saccharomyces boulardii*, a probiotic yeast. The aim is to develop multifunctional ingredients with enhanced bioactivity and techno‐functionality for use in plant‐based food applications, particularly beverages. Babassu mesocarp flour is rich in starch and phytochemicals with known antioxidant properties, whereas Brazil nuts are a source of proteins, unsaturated fats, and minerals, especially selenium. In addition to being underexplored substrates for fermentation, with significant regional economic value, these two raw materials have markedly different centesimal compositions, which presents a valuable opportunity to investigate how fermentation with *Saccharomyces boulardii* differentially modifies distinct food matrices (Rodrigues Sousa et al. 2023; Assmann et al. 2021).

The use of *S. boulardii* as starter culture in fermented products is justified by its probiotic properties, safety, and ability to withstand harsh gastrointestinal conditions. It contributes to fermentation by producing beneficial metabolites, enhancing the nutritional and sensory qualities of the final product. Moreover, its documented health benefits, including modulation of gut microbiota, anti‐inflammatory effects, and prevention of gastrointestinal infections, make it an attractive candidate for functional food formulations aiming to promote gut health (Gutiérrez‐Nava et al. 2024; dos Santos et al. 2023).

To assess the potential of these flours and the impact of *S. boulardii* fermentation, both fermented and non‐fermented lyophilized powders were evaluated for their antioxidant and antidiabetic activities, as well as for their techno‐functional properties, including oil holding capacity (OHC), water holding capacity (WHC), emulsifying capacity (EC), and critical gelling concentration (CGC). Following small‐scale fermentation in Erlenmeyer flasks, bench‐scale fermentation was carried out in a stirred‐tank bioreactor to simulate more controlled and scalable conditions, allowing for comparative analysis of process performance at different production scales.

To the best of our knowledge, no previous studies have investigated the fermentation of babassu or Brazil nut flours using *S. boulardii* as a probiotic agent. This represents a relevant research gap, considering the promising nutritional and functional potential of these raw materials and their underutilization in biotechnological applications. The results of this study offer valuable insights into the development of multifunctional, clean‐label ingredients for plant‐based beverages, while also contributing to the valorization of Amazonian plant resources.

## 2 Materials and Methods

### 2.1 Reagents

The reagents 6‐hydroxy‐2,5,7,8‐tetramethyl‐3,4‐dihydrochromene‐2‐carboxylic acid (Trolox), 2,2′‐azino‐bis(3‐ethylbenzothiazoline‐6‐sulfonic acid) (ABTS)^•+^, 2,2‐difenil‐1‐picrilhidrazil (DPPH), TPTZ, ρNPG, pancreatin from porcine pancreas, and α‐glucosidase from *Saccharomyces cerevisiae* were purchased from Sigma–Aldrich (Steinheim, Germany). Babassu mesocarp flour and Brazil nut flour were purchased from “Vem do Xingu Rede de Cantinas” (Altamira, Pará, Brazil). The flours were stored under vacuum at −20°C. The lyophilized yeast strain *Saccharomyces boulardii*‐17 (Repoflor 200) was purchased in capsule form from EMS Sigma Pharma (Hortolândia, Brazil). Other reagents used were of analytical grade.

### 2.2 Overview of Work

**FIGURE 1:**
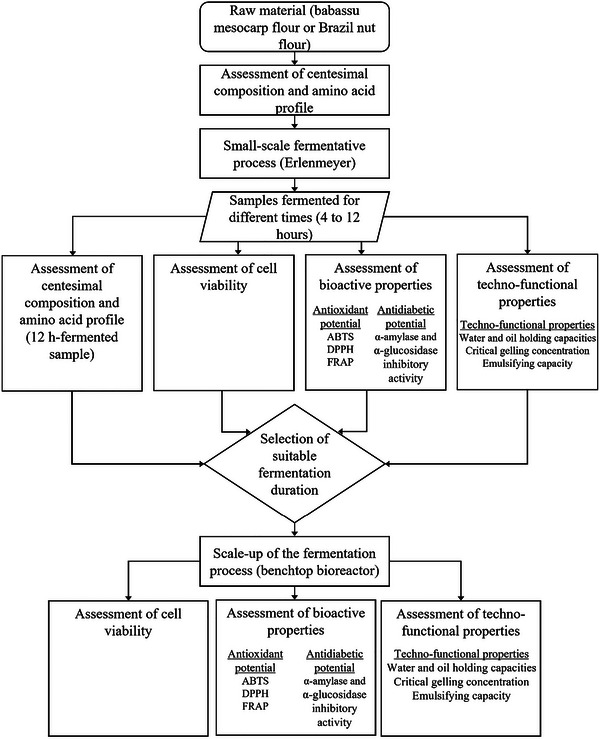
Experimental workflow for the development and characterization of fermented flours from babassu mesocarp and Brazil nut.

An overview of the steps executed in this study to obtain and characterize the fermented products, as described in the subsequent sections, is depicted in Figure 1.

### 2.3 Centesimal Composition and Amino Acid Profile

Moisture, ash, protein, and lipid contents were determined according to the Official Methods of Analysis of AOAC International (AOAC 2005). Moisture content was determined by drying the samples at 105°C to constant weight (AOAC 925.10). Ash content was determined by incineration in a muffle furnace at 550°C (AOAC 923.03). Crude protein was quantified using the Kjeldahl method (AOAC 2001.11), applying a nitrogen‐to‐protein conversion factor of 6.25. Lipid content was measured by Soxhlet extraction using petroleum ether as solvent (AOAC 963.15). Total carbohydrate content was estimated by difference, subtracting the sum of moisture, ash, protein, and lipid contents from the total weight of the sample.

Amino acid content was determined by high‐performance liquid chromatography (HPLC), following the methodology described by de Carvalho Cavenaghi et al. (2025). Prior to analysis, amino acids were released from the polypeptide chains through acid hydrolysis with 6 N HCl at 110°C for 24 h. For tryptophan determination, however, alkaline hydrolysis (typically using NaOH) was employed due to its degradation under acidic conditions.

### 2.4 Small‐Scale Fermentation Process

The lyophilized *S. boulardii*‐17 (Repoflor 200, Legrand Pharma, Brazil) was used as the starter culture. For yeast activation and pre‐inoculum preparation, the contents of one capsule were dispersed in YEPD medium (1% w/v yeast extract; 2% w/v peptone; and 2% w/v dextrose) and cultivated for three consecutive generations at 30°C at 150 rpm, with each generation incubated for 24 h. The fermentation of the flours was carried out following these steps: (i) substrate preparation (10 g of flour placed in 250 mL Erlenmeyer flasks, autoclaved at 121°C for 15 min, and dispersed in 100 mL of sterile distilled water), (ii) inoculation of the activated yeast (2% of the pre‐inoculum at a concentration of 10^8^ cells mL^−1^), and (iii) incubation at 30°C under orbital agitation (TE‐424, Tecnal Equipamentos Científicos, Piracicaba, Brazil) at 150 rpm. The fermentation process was carried out for 12 h, with samples collected every 4 h to evaluate the effect of fermentation duration on both bioactive and techno‐functional properties. The samples obtained after babassu mesocarp and Brazil nut flour fermentation for 4, 8, and 12 h were labeled as follows: FBMF‐4 h, FBMF‐8 h, FBMF‐12 h, FBNF‐4 h, FBNF‐8 h, and FBNF‐12 h, respectively. The samples collected were frozen and lyophilized (Liotop L101, Liobras, São Paulo, Brazil) for 48 h and stored at −20°C for further analysis. To determine viable cell count, the process was extended to 48 h, with samples being collected every 12 h after the initial 12 process hours.

Control samples were the non‐fermented autoclaved flours (NFBMF, non‐fermented babassu mesocarp flour and NFBNF, non‐fermented Brazil nut flour).

### 2.5 Viable Cell Count for Probiotic Potential Assessment

Each sample was serially diluted in YEPD broth and plated on YEPD agar using the drop‐plate technique and incubated at 30°C for 24 h. After the incubation period, the cells were counted and reported as the logarithm of cell‐forming units per milliliter (log_10_ CFU mL^−1^) Andrade and de Castro (2023).

### 2.6 Bioactive Properties

#### 2.6.1 Extract Preparation for Antioxidant Property Measurements

Extracts of babassu and Brazil nut flours were prepared by dispersing the control flour or lyophilized samples in distilled water at a concentration of 1 mg mL^−1^ (babassu) or 10 mg mL^−1^ (Brazil nut) and submitted to ultrasonication (50–60 Hz) for 10 min at room temperature, followed by centrifugation at 12,000 *g*, for 10 min at 5°C (Himac CR21GII, Hitachi, Ibaraki, Japan). The supernatants were then collected and used for determinations.

##### 2.6.1.1 ABTS^+^ Radical‐Scavenging Activity

The ABTS^+^ radical was generated by mixing 5 mL of ABTS solution (7 mmol L^−1^) with 88 µL of K_2_S_2_O_8_ (140 mmol L^−1^). The mixture was left at room temperature for 16 h in the dark to allow for complete radical formation. Prior to use, the ABTS^+^ solution was diluted to obtain an absorbance of 0.70 ± 0.02 at 734 nm. For the assay, 40 µL of each extract was mixed with 440 µL of the ABTS^+^ solution in Eppendorf tubes and incubated for 6 min at room temperature. A control reaction was prepared by replacing the sample with distilled water. All reactions were performed in triplicate. Absorbance was measured at 734 nm using a microplate reader (Multiskan GO, Thermo Fisher Scientific, Finland). Results were calculated using a Trolox standard curve (*y* = −0.0014*x* + 0.5339; *R* ^2^ = 0.99) and expressed as µmol of Trolox Equivalent per gram of flour (µmol TE g^−1^) (Rasera et al. 2023).

##### 2.6.1.2 DPPH Radical‐Scavenging Activity

The reaction mixture containing 132 µL of each extract and 268 µL of DPPH solution (150 mmol L^−1^) was mixed in Eppendorf tubes and incubated at room temperature for 45 min in the dark. A control reaction was prepared using distilled water instead of the sample. All reactions were conducted in triplicate. Absorbance was measured at 517 nm using a microplate reader (Multiskan GO, Thermo Fisher Scientific, Finland). Antioxidant activity was calculated using a Trolox standard curve (*y* = −0.0033*x* + 0.4663; *R* ^2^ = 0.99), and results were expressed as µmol of Trolox equivalents per gram of flour (µmol TE g^−1^) (de Matos et al. 2021).

##### 2.6.1.3 Ferric Reducing Antioxidant Power (FRAP)

The FRAP reagent was prepared by mixing TPTZ (10 mmol L^−1^, and dissolved in 40 mmol L^−1^ HCl), FeCl_3_·6H_2_O (20 mmol L^−1^) and acetate buffer (0.3 mol L^−1^, and pH 3.6) in a 1:1:10 ratio, respectively. Then, 50 µL of each extract was combined with 350 µL of the freshly prepared FRAP reagent in Eppendorf tubes and incubated at 37°C for 30 min. A control reaction was prepared using distilled water instead of the sample. All reactions were performed in triplicate. Absorbance was measured at 595 nm using a microplate reader (Multiskan GO, Thermo Fisher Scientific, Finland). Antioxidant capacity was calculated using a Trolox standard curve (*y* = 0.0025*x* + 0.0513; *R* ^2^ = 0.99), and results were expressed as µmol of Trolox equivalents per gram of flour (µmol TE g^−1^) (de Matos et al. 2024).

#### 2.6.2 Determination of In Vitro Antidiabetic Properties

##### 2.6.2.1 α‐Amylase Inhibitory Activity

The reaction mixture containing 40 µL of porcine pancreatin enzyme solution (0.5 mg mL^−1^ in 0.1 mol L^−1^ phosphate buffer at pH 7.0) and 40 µL of extracts was incubated for 30 min at 37°C. Subsequently, 40 µL of a 0.1% (w/v) starch solution was added to the mixture, which was kept at 37°C for 10 min. The reaction was stopped with 20 µL of HCl, and 100 µL of 5 mmol L^−1^ of iodine solution was added. The absorbance was read at 580 nm using a microplate reader (Multiskan GO, Thermo Fisher Scientific). A control without enzyme was prepared by replacing the sample and the enzymatic solution with phosphate buffer, and a control with enzyme was also prepared by replacing only the sample solution with buffer solution. To eliminate potential interference from residual starch in the samples, a blank test was prepared by replacing the enzyme solution with buffer. A negative control was prepared by replacing the sample with DMSO, whereas acarbose served as the positive control and was tested at concentrations of 1.0, 5.0, and 10 mg mL^−1^ (Dra et al. 2019). The inhibitory activity of α‐amylase (%) was calculated according to the following equation:

(1) $$\mathrm{Inhibition}\;\left(\%\right)=\frac{\mathrm{Ab}s_{\mathrm{sample}}-\mathrm{Ab}s_{\mathrm{control}\;\mathrm{with}\;\mathrm{enzyme}}}{\mathrm{Ab}s_{\mathrm{control}\;\mathrm{without}\;\mathrm{enzyme}}}\times 100$$

##### 2.6.2.2 α‐Glucosidase Inhibitory Activity

The reaction mixture containing 50 µL of extracts and 100 µL of enzymatic solution (α‐glucosidase from *S. cerevisiae*, at a concentration of 0.1 U mL^−1^) was incubated at 37°C for 10 min. Then, 50 µL of substrate (5 mmol L^−1^ *p*‐nitrophenyl‐α‐d‐glucopyranoside solution) was added to the reaction mixture and kept at 37°C for 5 min. Absorbance was read using a microplate reader (Multiskan GO, Thermo Fisher Scientific) at 405 nm. The control reaction was prepared by replacing the sample with phosphate buffer in the reaction mixture (de Matos et al. 2022; Apostolidis et al. 2007). The inhibitory activity of α‐glucosidase (%) was calculated according to the following equation:

(2) $$\mathrm{Inhibition}\;\left(\%\right)=\frac{\mathrm{Ab}s_{\mathrm{control}}-\mathrm{Ab}s_{\mathrm{sample}}}{\mathrm{Ab}s_{\mathrm{control}}}\times 100$$

### 2.7 Techno‐Functional Properties

#### 2.7.1 Water and Oil Holding Capacities

To evaluate the WHC and OHC, approximately 100 mg of flour samples were placed in Eppendorf microtubes. The mass of each microtube was previously weighed. Then, 1 mL of distilled water (for water absorption capacity) or 1 mL of soybean oil (for oil absorption capacity) was added to the microtubes, and the suspensions were then vortexed for 1 min and kept at rest for 30 min at room temperature. The microtubes were then centrifuged at room temperature at 16,000 *g* for 20 min (Himac CR21GII, Hitachi). The supernatant was discarded, the residual supernatant was removed from the edges of the microtubes with absorbent paper, and the microtube with the precipitate was weighed again (Silva et al. 2022). The WHC or OHC (g g^−1^) was calculated according to the following equation:

(3) $$\mathrm{WHC}\;\mathrm{or}\;\mathrm{OHC}\;\left(g\;g^{-1}\right)=\frac{M_{1}-M_{t}-M_{0}}{M_{0}}$$

where *M*_1_ is the mass (g) of the tube containing the wet sample after discarding the residual water or oil supernatant, *M*_0_ is the initial mass (g) of the sample, and *M_t_* is the mass (g) of the Eppendorf microtube.

#### 2.7.2 Critical Gelling Concentration

Aliquots of 5 mL from flour samples at different concentrations (0.02–0.2 g mL^−1^) were transferred to 30 mL test tubes and suspended in Milli‐Q water. The mixtures were vortexed for 1 min and then heated in a water bath at 100°C for 60 min. After incubation, the samples were immediately cooled in an ice bath and subsequently refrigerated at 4°C for 2 h. To assess gel formation, the tubes were slowly inverted to check for flow. The CGC (CMC, g mL^−1^) was defined as the lowest concentration at which the sample did not flow upon tube inversion (Silva et al. 2022).

#### 2.7.3 Emulsifying Capacity

The flour samples, at a concentration of 0.2% (w/v), were dispersed in 0.075% NaCl (w/v), and 20% (v/v) of soybean oil was then added to the dispersion. The mixture was stirred in an Ultra Turrax disperser for 2 min at 9000 rpm. Immediately after emulsion formation, an aliquot of 1.5 mL was placed in Eppendorf microtubes and centrifuged at 4°C at 1200 *g* for 10 min (Himac CR21GII, Hitachi) (Pérez‐Andrés et al. 2019). The amount of free oil (supernatant) was weighed, and the EC (%) was calculated as described in the following equation:

(4) $$\mathrm{Emulsifying}\;\mathrm{capacity}\;\left(\%\right)=\left(1-\;\frac{g_{e}\times \rho_{e}}{V_{a}}\right)\times 100$$

where *g_e_* is the mass of free oil (g), *ρ_e_* is the density of soybean oil (g mL^−1^), and *V_a_* is the volume of oil added (mL).

### 2.8 Scale‐Up of the Fermentation Process in a Benchtop Bioreactor

The scale‐up of the fermentation process was conducted in a 6 L capacity benchtop bioreactor with a working volume of 3 L (Bioflo‐IIc, New Brunswick Co., USA). The fermented products obtained in the scale‐up studies (FBMS‐12 h—babassu mesocarp flour fermented in a bioreactor for 12 h and FBNS‐12 h—Brazil nut flour fermented in a bioreactor for 12 h) were evaluated regarding the previously described methods for viable cell count, antioxidant and antidiabetic potentials, and techno‐functionalities (Sections 2.5–2.7). The centesimal composition was also assessed, as described in Section 2.3. Additionally, for antioxidant and antidiabetic properties, the determination of IC_50_ was also performed—the concentration (mg mL^−1^) required to inhibit 50% of radicals (antioxidant activities) or enzymes (antidiabetic activities). IC_50_ values were determined by evaluating the ABTS and DPPH radical‐scavenging activities of babassu and Brazil nut flours at various concentrations. Control assays were performed using distilled water in place of the sample. The analytical curves generated by plotting percentage inhibition against concentration were used to calculate the IC_50_. For babassu flour, concentrations ranged from 0.2 to 2.0 mg mL^−1^ for the ABTS assay (*y* = 33.806*x* + 0.1842; *R* ^2^ = 0.99) and from 0.2 to 1.2 mg mL^−1^ for the DPPH assay (*y* = 73.022*x* + 0.9719; *R* ^2^ = 0.99). For Brazil nut flour, the concentration range was 5.0–25.0 mg mL^−1^ for the ABTS assay (*y* = 2.5458*x* + 0.6263; *R* ^2^ = 0.99) and 5.0 to 18.0 mg mL^−1^ for the DPPH assay (*y* = 3.0518*x* + 0.1534; *R* ^2^ = 0.99). For the antidiabetic assays, the concentration of babassu flour ranged from 0.2 to 0.8 mg mL^−1^ for α‐glucosidase inhibition (*y* = 103.51*x* − 1.2407; *R* ^2^ = 0.98) and from 1.2 to 5.0 for α‐amylase inhibition (*y* = 17.511 − 15.149; *R* ^2^ = 0.97). The IC_50_ values for each determination, representing the concentration required to achieve 50% inhibition of free radicals in antioxidant activity assays or enzyme inhibition in assays related to antidiabetic properties, were calculated by interpolation of the concentration–response curves.

### 2.9 Statistical Analyses

Analysis of variance (ANOVA) followed by Tukey's post hoc test was conducted using Minitab 19 software (Minitab Inc., Pennsylvania, USA), with a significance level set at 5% (*p* < 0.05). All analyses were performed in triplicate, and the results were presented as mean ± standard deviation.

## 3 Results and Discussion

### 3.1 Composition and Amino Acid Profile

The centesimal compositions of the raw and fermented babassu mesocarp and Brazil nut flours are shown in Table 1. The flour obtained from babassu kernels is high in carbohydrates (96.41 g per 100 g) and low in lipid and protein. NFBNF flour is characterized by its high lipid content (44.62 g per 100 g) and protein (33.65 g per 100 g).

**TABLE 1:** Centesimal composition and amino acid profile, on a dry weight basis per 100 g of babassu and Brazil nut flours (raw or fermented for 12 h).

Parameters	Characteristic	NFBMF	FBMF‐12 h	Variation (%)	NFBNF	FBNF‐12 h	Variation (%)
**Carbohydrate**	—	96.41^a^ ± 0.25	96.63^a^ ± 0.07	0.23	15.03^a^ ± 0.62	14.06^b^ ± 0.55	−6.47
**Protein**	—	1.77^b^ ± 0.07	2.17^a^ ± 0.06	22.24	33.65^a^ ± 0.21	33.68^a^ ± 0.12	0.09
**Fat**	—	0.64^a^ ± 0.17	0.29^b^ ± 0.06	−54.02	44.62^a^ ± 0.87	45.77^a^ ± 0.53	2.59
**Ashes**	—	1.18^a^ ± 0.04	0.91^b^ ± 0.07	−23.05	6.70^a^ ± 0.05	6.49^a^ ± 0.04	−3.17
**N‐EAA**							
Aspartic acid + asparagine	Acidic	0.09^b^ ± 0.00	0.24^a^ ± 0.00	166.67	2.31^a^ ± 0.04	2.40^a^ ± 0.04	3.90
Glutamic acid + glutamine	Acidic	0.09^b^ ± 0.00	0.28^a^ ± 0.00	211.11	5.75^b^ ± 0.11	6.31^a^ ± 0.12	9.74
Serine	Neutral‐polar	0.04^b^ ± 0.00	0.10^a^ ± 0.00	150.00	1.23^a^ ± 0.02	1.23^a^ ± 0.02	0.00
Glycine	Neutral‐polar	0.08^b^ ± 0.00	0.23^a^ ± 0.00	187.50	1.32^b^ ± 0.02	1.44^a^ ± 0.03	9.09
Histidine	Basic	<0.01	<0.01	—	0.63^a^ ± 0.01	0.63^a^ ± 0.01	0.00
Arginine	Basic	<0.01	0.16 ± 0.00	—	4.22^a^ ± 0.08	4.01^b^ ± 0.07	−4.98
Alanine	Basic	0.13^b^ ± 0.00	0.17^a^ ± 0.00	30.77	1.09^b^ ± 0.02	1.14^a^ ± 0.02	4.59
Proline	Neutral‐nonpolar	0.07^b^ ± 0.00	0.27^a^ ± 0.00	285.71	1.27^b^ ± 0.02	1.37^a^ ± 0.03	7.87
Tyrosine	Neutral‐polar	0.09^b^ ± 0.00	0.14^a^ ± 0.00	55.56	0.82^b^ ± 0.02	0.86^a^ ± 0.02	4.88
Cysteine	Neutral‐polar	<0.01	<0.01	—	0.56^b^ ± 0.01	0.65^a^ ± 0.01	16.07
Hydroxyproline	Polar	0.03^b^ ± 0.00	0.22^a^ ± 0.00	633.33	0.03^b^ ± 0.00	0.22^a^ ± 0.00	633.33
**EAA**							
Threonine	Neutral‐polar	<0.01	0.06 ± 0.00	—	0.79^a^ ± 0.01	0.82^a^ ± 0.02	3.80
Valine	Neutral‐nonpolar	0.06^b^ ± 0.00	0.14^a^ ± 0.00	133.33	1.29^b^ ± 0.02	1.42^a^ ± 0.03	10.08
Methionine	Neutral‐nonpolar	<0.01	0.04 ± 0.00	—	2.01^a^ ± 0.04	1.95^a^ ± 0.04	−2.99
Isoleucine	Neutral‐nonpolar	0.05^b^ ± 0.00	0.11^a^ ± 0.00	120.00	0.90^b^ ± 0.02	0.98^a^ ± 0.02	8.89
Leucine	Neutral‐nonpolar	0.24^a^ ± 0.01	0.18^b^ ± 0.00	−25.00	2.18^a^ ± 0.04	2.25^a^ ± 0.04	3.21
Phenylalanine	Neutral‐nonpolar	0.06^b^ ± 0.00	0.15^a^ ± 0.00	150.00	1.08^b^ ± 0.02	1.20^a^ ± 0.02	11.11
Lysine	Neutral‐nonpolar	0.07^b^ ± 0.00	0.15^a^ ± 0.00	114.29	0.93^b^ ± 0.02	0.97^a^ ± 0.02	4.30
Tryptophan	Nonpolar	0.15^a^ ± 0.00	0.14^b^ ± 0.00	−6.67	0.43^a^ ± 0.01	0.35^b^ ± 0.01	−18.60
**Total amino acids**		1.26^b^ ± 0.01	2.63^a^ ± 0.02^a^	108.73	28.84^a^ ± 0.53	29.96^a^ ± 0.56	3.88

*Note*: Results are presented as mean (*n* = 3) ± standard deviation. Different superscript letters indicate significant differences (*p* < 0.05) for a given amino acid within the same type of substrate, fermented or not. NFBMF = non‐fermented babassu mesocarp flour; FBMFS‐12 h = babassu mesocarp flour fermented for 12 h; NFBNF = non‐fermented Brazil nut flour; FBNFS‐12 h = Brazil nut flour fermented for 12 h. N‐EAA = non‐essential amino acids. EAA = essential amino acids. Percentual variation was calculated using the amino acid content of fermented sample in relation to the non‐fermented sample.

The composition of babassu (FBMF) and Brazil nut (FBNF) flours showed distinct responses to fermentation with *S. cerevisiae* var. *boulardii*, which can be attributed to the markedly different composition of the two substrates. Babassu flour is composed predominantly of carbohydrates, particularly starch, and contains low levels of protein, lipid, and ash. In this matrix, the growth of *S. boulardii* led to statistically significant (*p* ≤ 0.05) changes, even when the absolute variations were small. The increase in babassu protein content (22.24%) may be attributed to the accumulation of microbial biomass, as reported by Li et al. (2025). The observed variation may also be attributed to a reduction in other components, such as fats, due to the hydrolytic activity of enzymes released by the microorganism to obtain energy—rather than reflecting a true increase in protein content, as suggested by Nkhata et al. (2018).

The significant reduction in fat content (−54.02%) suggests that lipids may have been metabolized by the yeast as an energy source to support growth and maintenance, as previously observed in other fermentations involving lipid‐containing substrates. A similar trend was observed by Madeira et al. (2025) in fermented beverages formulated with babassu, coconut, and cashew apple. According to this study, this reduction may be associated with a decrease in saturated fatty acids, as these compounds are utilized by probiotic microorganisms for the synthesis of cellular structures, including the cell membrane and wall of lactic acid bacteria.

A noticeable decrease in ash content (−23.05%) was also observed in babassu flour after fermentation. This trend aligns with findings from a study on a symbiotic drink formulated with Brazil nuts and fermented with *Lactobacillus casei*, where a 38.5% reduction in ash content was reported after 12 h of fermentation (da Cunha Júnior et al. 2020). Such reductions may be attributed to microbial utilization of minerals to support growth and metabolism, as well as potential alterations in the solubility or extractability of mineral compounds during the fermentation process.

In contrast, Brazil nut flour, which is naturally rich in proteins and lipids, showed minimal compositional changes after fermentation. The abundance of these macronutrients likely limited detectable variations, as the microbial demand may not have been sufficient to significantly alter their levels. The only statistically significant difference observed (*p* < 0.05) was a reduction in carbohydrate content, consistent with the findings of da Cunha Júnior et al. (2020). This reduction is likely due to hydrolysis and subsequent utilization of carbohydrates by the yeast as an energy source to support its metabolic activity and growth.

The amino acid profile of non‐fermented and 12‐h fermented flour is presented in Table 1. The main amino acids present in Brazil nut flour were Glu (5.75%), Arg (4.22%), Asp (2.31%), and Leu (2.18%), in that order. Furthermore, Brazil nut flour contains all EAA (33.32% of total amino acid content). Babassu flour, on the other hand, had a smaller variety of amino acids that were detected in lower quantities, which was expected due to its low protein content. The most abundant amino acids in babassu flour were Leu (0.24%), Trp (0.15%), and Ala (0.13%). Babassu's EAA corresponds to 50% of total amino acid content.

Fermentation positively affected the amino acid composition of FBMF‐12 h, leading to significant increases (*p* < 0.05) in both the quantity and diversity of amino acids, except for Leu and Trp. Significant increases were also observed in Glu, Gly, Ala, Pro, Tyr, Cys, Hyp, Val, Iso, Phe, and Lys content in FBNFS‐12 h. Demirgul et al. (2022) assessed the amino acid composition of yeast extracts and found that Glu is among the most prevalent amino acids, suggesting that the modifications in amino acid content are mostly related to biomass growth. This indicates that some amino acids are utilized for cell proliferation, whereas others are produced as metabolic byproducts or derivatives of yeast cells.

The amino acid composition of the substrate is one of the key factors that regulate microbial growth. Except for Cys, His, and Lys, yeasts can use all l‐amino acids as the sole nitrogen source (Takagi 2019; Ljungdahl and Daignan‐Fornier 2012). Godard et al. (2007) investigated the gene expression profile of *Saccharomyces boulardii* when grown in media containing different nitrogen sources. They found that amino acid uptake is regulated by the Ssy1‐Ptr3‐Ssy5 (SPS) sensing pathway, which activates the expression of specific amino acid permeases in response to the type of extracellular amino acids present in the medium. The SPS sensor's response varies depending on the amino acid available. Seven amino acids—Asn, Gln, Ser, Asp, Ala, Arg, and Glu—were identified as “Class A” or preferred nitrogen sources, as they supported the shortest generation times. These amino acids are favored by the cells because they provide carbon skeletons that are readily integrated into central metabolic pathways. Given the concentration of flour added to the culture medium, 35 mg of these seven amino acids were available for microorganism cultivation using babassu as substrate (13% of total amino acid) compared to 1592 mg available when using Brazil nut (55% of total amino acid) (Table 1). This could further explain why cell growth was maximized for Brazil nut.

### 3.2 Growth Kinetics of *S. boulardii* in Babassu and Brazil Nut‐Based Media

**FIGURE 2:**
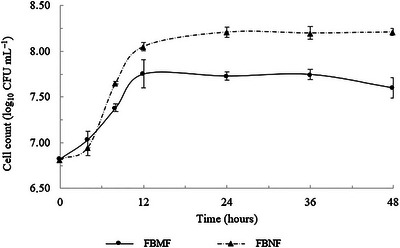
Growth of *Saccharomyces boulardii*‐17 during a 48‐h submerged fermentation process. Cell counts are presented as mean (*n* = 3), with error bars representing the standard deviation. FBMF, fermented babassu mesocarp flour; FBNF, fermented Brazil nut flour.

The growth profile of *S. boulardii*‐17 in babassu and Brazil nut flours is shown in Figure 2. The yeast was able to proliferate in both substrates, reaching its maximum cell concentration at 12 h of fermentation, after which growth plateaued, indicating entry into the stationary phase. However, a higher final cell count was observed when Brazil nut flour was used (8.05 log_10_ CFU mL^−1^) when compared to babassu flour (7.73 log_10_ CFU mL^−1^). This enhanced microbial growth can be attributed to the more favorable nutritional profile of Brazil nut flour. Unlike babassu, which is predominantly composed of starch—a polysaccharide that *S. boulardii* cannot metabolize due to the absence of amylolytic enzymes, such as α‐amylase and glucoamylase (Aydemir 2014; Latorre‐García et al. 2005)—Brazil nut flour contains higher levels of proteins and lipids, both of which can serve as alternative sources of carbon and nitrogen for yeast metabolism.

Furthermore, Brazil nuts are known to be rich in selenium, magnesium, and vitamin E (Alcântara et al. 2024; Assmann et al. 2021), micronutrients that may enhance yeast growth and viability by supporting cellular metabolism and oxidative stress defense. The yeast might have encountered a more readily assimilable pool of nutrients, allowing for more efficient biomass accumulation within a shorter time frame. Taken together, the superior growth performance of *S. boulardii*‐17 in Brazil nut flour underscores the importance of nutrient composition, especially the balance between carbon, nitrogen, and micronutrients, in supporting yeast proliferation during fermentation.

However, babassu is rich in phenolic compounds, considered a prebiotic compound for improving microorganism growth, and may have contributed to the improvement in the number of *S. boulardii*‐17 cells in the fermented babassu samples (Liu et al. 2022). Cell growth reached the maximum value of log_10_ CFU mL^−1^ after 12 h of fermentation for both flours, entering the stationary phase (where there is no further cell multiplication) after this period.

Considering that most probiotic food matrices are traditionally dairy‐based and that there is a growing demand for alternatives among consumers with specific dietary restrictions—such as dietary preferences excluding animal products and individuals with lactose intolerance or milk allergies (Cosme et al. 2022), the use of plant‐based substrates like babassu and Brazil nut flour represents a promising strategy for developing innovative, non‐dairy probiotic carrier foods. These substrates offer not only nutritional value but also align with current trends toward more inclusive and sustainable food systems.

### 3.3 Bioactive Properties

#### 3.3.1 In Vitro Antioxidant Potential

Fermentation had a significant and positive impact (*p* < 0.05) on the antioxidant activity measured by FRAP assay for babassu samples; the highest activity was obtained after 12 h, with 181.77 µmol TE g^−1^ (Table 2). For the ABTS and DPPH methods, the highest antioxidant activities were also observed after 12 h of fermentation (92.76 and 105.07 µmol TE g^−1^, respectively), although these results do not differ statistically (*p* > 0.05) from those observed for NFBMF. For babassu samples, in all assays, an initial reduction in the antioxidant potential after 4 h of fermentation was detected, followed by a subsequent increase. This initial reduction may be related to the metabolization of bioactive compounds and their use as a nutrient source by the microorganism; afterwards, the antioxidant activity increased due to the release of antioxidant compounds from yeast metabolism or the biotransformation of the substrate matrix, such as the release of bound phenolics, peptides, and amino acids (da Cruz et al. 2023).

**TABLE 2:** Antioxidant potential of babassu mesocarp flour and Brazil nut flour before and after fermentation with *Saccharomyces boulardii*‐17.

Sample	ABTS (µmol TE g^−1^)	DPPH (µmol TE g^−1^)	FRAP (µmol TE g^−1^)
**NFBMF**	75.00^ab^ ± 13.66	101.22^a^ ± 4.62	168.42^b^ ± 3.39
**FBMF‐4 h**	68.4^b^ ± 4.02	77.68^b^ ± 11.27	139.08^c^ ± 3.97
**FBMF‐8 h**	80.60^ab^ ± 3.60	96.55^a^ ± 0.01	139.08^c^ ± 3.91
**FBMF‐12 h**	92.76^a^ ± 1.26	105.07^a^ ± 0.01	181.77^a^ ± 4.46
**NFBNF**	21.13^a^ ± 4.48	5.43^b^ ± 0.22	8.32^b^ ± 0.62
**FBNF‐4 h**	14.82^b^ ± 1.24	6.48^a^ ± 0.22	11.77^a^ ± 0.56
**FBNF‐8 h**	17.09^ab^ ± 0.97	6.15^a^ ± 0.12	7.76^b^ ± 0.66
**FBNF‐12 h**	17.01^ab^ ± 0.29	6.25^a^ ± 0.09	7.27^b^ ± 0.32

*Note*: Results presented as mean (*n* = 3) ± standard deviation. Different superscript letters indicate significant differences (*p* < 0.05) between samples in the same column (for one type of substrate). NFBMF = non‐fermented babassu mesocarp flour; FBMF‐4 h, FBMF‐8 h, and FBMF‐12 h = babassu mesocarp flour fermented for 4, 8, and 12 h, respectively; NFBNF = non‐fermented Brazil nut flour; FBNF‐4 h, FBNF‐8 h, and FBNF‐12 h = Brazil nut flour fermented for 4, 8, and 12 h, respectively.

Abbreviations: ABTS, 2,2′‐azino‐bis(3‐ethylbenzothiazoline‐6‐sulfonic acid); DPPH, 2,2‐difenil‐1‐picrilhidrazil; FRAP, ferric reducing antioxidant power.

Fermentation also increased the antioxidant activity of Brazil nut flour measured by DPPH and FRAP methods. The highest values obtained were 6.48 and 19.34 µmol TE g^−1^, respectively, with both found in FBNF‐4 h and statistically (*p* < 0.05) higher than the ones observed for NFBNF (5.43 µmol TE g^−1^ for DPPH and 8.32 µmol TE g^−1^ for FRAP). Conversely, NFBNF exhibited the highest ABTS^+^ radical‐scavenging activity (21.13 µmol TE g^−1^), although no significant difference (*p* > 0.05) was found between this sample and FBNF‐8 h or FBNF‐12 h.

The antioxidant activity of vegetal matrices is abundantly reported in scientific literature and is associated with the presence of bioactive compounds, such as phenolic compounds and flavonoids (Lima et al. 2023). During yeast fermentation, a series of transformations in the structure of phenolic compounds or their content can happen, such as the release of insoluble compounds bound to the cell wall and oxidation and hydrolysis that can improve, decrease, or not affect the antioxidant activities (Martínez‐Mendoza et al. 2023).

The antioxidant potential of fermented products can also be affected by metabolites produced by *S. boulardii*. The global metabolite profile of *S. cerevisiae* var. *boulardii* NCYC 3264 was investigated by Datta et al. (2017), and among the 22 extracellular metabolites and 18 intracellular metabolites found, many are reported to act as antioxidants, like vitamin B_6_, organic acids (vanillic, citric, and cinnamic acids), and 2,4‐bis(1,1‐dimethylethyl)‐phenol (antioxidant no. 33). *S. cerevisiae* extracts also contain significative amounts of phenolics, notably caffeic and gallic acid (Demirgul et al. 2022). Cell supernatants of *S. boulardii* were found to have DPPH radical‐scavenging activities (up to 67.0%), which further corroborates the antioxidant properties of this yeast (Fu et al. 2023).

Antioxidant amino acids and peptides, naturally present in babassu and Brazil nut, released from the food matrix during fermentation due to the proteolytic action of *S. boulardii*, or released from its biomass, also have an important contribution to the antioxidant activity observed in the samples analyzed in this study. Demirgul et al. (2022) highlight that certain yeast strains can synthesize glutathione, a linear tripeptide (Glu–Cys–Gly) known for its powerful antioxidant activity. Due to high protein content, the effect of amino acid composition on the antioxidant activity of Brazil nut samples is worthy of a deeper investigation.

Amino acids can inhibit oxidation through various mechanisms like free radical scavenging, metal chelation, and inactivation of reactive oxygen species. Guidea et al. (2020) evaluated the antioxidant activity of 20 free amino acids. Asp, Cys, Glu, Ile, and Tyr were able to scavenge more than 80% of DPPH radical at a concentration of 5 mM. The authors emphasized that amino acids containing aromatic moieties (Tyr) or heteroatoms (Asp, Cys, Glu) in their side chains seem to have satisfactory DPPH scavenging activity. Fermentation of Brazil nut samples had a positive impact on the content of all four of these amino acids (Table 1), which may help explain the 15.10% increase in DPPH scavenging activity of FBMF‐12 h in comparison to NFBMF (Table 2). The same study observed that the scavenging activity of ABTS radical was generally lower for the amino acids evaluated, with Cys being the only one with strong activity. In our study, for this method, no significant difference (*p* < 0.05) was found between NFBMF and FBMF‐12 h. Cys and Trp were the amino acids with the best results observed for the FRAP method, accounting for 96.72% and 85.30% of reducing power, respectively. The authors underscored that the reduction power of these amino acids is attributed to the modulation of redox reactions by thiol groups (–SH) of Cys and the indole group (NH) of Trp.

The effect of fermentation with *S. boulardii* on the antioxidant properties of vegetable food matrices has been reported in literature before. Shao et al. (2022) evaluated the effect of *S. boulardii* fermentation on Chinese yam. The results showed a positive effect for the process to scavenge the DPPH radical of the fermented extract (FCYP, IC_50_ = 1.76 mg mL^−1^), compared to the non‐fermented (UCYP, IC_50_ = 2.50 mg mL^−1^). The scavenging of the ABTS^+^ radical was also slightly increased by fermentation (IC_50_ = 18.18 mg mL^−1^ for FCYP against 18.51 mg mL^−1^ for UCYP). The authors attribute the improvement of the antioxidant activity to hydrogen‐donating metabolites synthetized by *S. boulardii* or to polysaccharides that were degraded by the microorganism, because DPPH scavenging activity is based on electron transfer (ET), and ABTS^+^ scavenging is based on both hydrogen atom transfer (HAT) and ET reactions.

Martínez‐Mendoza et al. (2023) found that passion fruit pulp fermented with *S. boulardii* CNCM I‐745 for 30 h showed a reduction in both DPPH scavenging activity (IC_50_, mg eq. Trolox mL^−1^ = 0.011) and FRAP activity (0.200 mg eq. Trolox mL^−1^) compared to the negative control, the pulp without yeast inoculation (IC_50_ = 0.007 mg eq. Trolox mL^−1^ for DPPH and 0.628 mg eq. Trolox mL^−1^ for FRAP), although an improvement in the ABTS scavenging activity was observed. The same study found a reduction in the DPPH scavenging activity for the fermented soursop pulp (IC_50_, mg eq. Trolox mL^−1^ = 0.030) compared to the non‐inoculated control sample (IC_50_, mg eq. Trolox mL^−1^ = 0.020). Phenolic compounds, vitamin C, and carotenoid levels increased after fermentation, and the explanation for the decrease in antioxidant capacity remains unclear, although alterations in other unmeasured components may also be related to the results observed.

#### 3.3.2 In Vitro Antidiabetic Potential

Individuals with type 2 diabetes mellitus may suffer postprandial hyperglycemia as a consequence of a reduction in insulin sensitivity, which can lead to various deleterious effects such as oxidative stress, production of advanced glycation end products (AGEs), and atherosclerosis (Hiyoshi et al. 2019). The increase in blood glucose levels is a result of the action of α‐amylase and α‐glucosidase, digestive enzymes that hydrolyze starch and oligosaccharides/disaccharides, respectively. The use of substances with inhibitory activity against these enzymes is a strategy to slow down absorption of carbohydrates and control postprandial hyperglycemia (Farias et al. 2021; Li et al. 2018).

In this work, babassu and Brazil nut samples showed antidiabetic potential, with babassu extracts exhibiting the most promising results (Table 3). A similar pattern to what was observed in antioxidant assays for babassu flours was seen for α‐amylase inhibition, in which the first hours of fermentation promote a decrease in the inhibitory activity, followed by an increase in the final hours of process. After 12 h of fermentation, the inhibitory activity of FBMF‐12 h (65.11%) is statistically equivalent (*p* > 0.05) to NFBMF extracts (68.61%), the highest value obtained. FBMF‐8 h showed the highest inhibition of α‐glucosidase, reaching 40.26%.

**TABLE 3:** Antidiabetic potential of babassu mesocarp flour and Brazil nut flour before and after fermentation with *Saccharomyces boulardii*‐17.

Sample	α‐Amylase inhibition (%)	α‐Glucosidase inhibition (%)
**NFBMF**	68.61^a^ ± 3.26	33.05^b^ ± 1.37
**FBMF‐4 h**	46.58^b^ ± 1.96	30.97^b^ ± 0.82
**FBMF‐8 h**	34.14^c^ ± 2.77	40.26^a^ ± 4.55
**FBMF‐12 h**	65.11^a^ ± 3.37	35.87^ab^ ± 1.07
**NFBNF**	27.06^a^ ± 1.08	Not detected
**FBNF‐4 h**	23.34^b^ ± 1.45	
**FBNF‐8 h**	19.99^c^ ± 1.35	
**FBNF‐12 h**	16.59^d^ ± 1.08	

*Note*: Results presented as mean (*n* = 3) ± standard deviation. Different letters indicate significant differences (*p* < 0.05) between samples in the same column (for one type of substrate). NFBMF = non‐fermented babassu mesocarp flour; FBMF‐4 h, FBMF‐8 h, and FBMF‐12 h = babassu mesocarp flour fermented for 4, 8, and 12 h, respectively; NFBNF = non‐fermented Brazil nut flour; FBNF‐4 h, FBNF‐8 h, and FBNF‐12 h = Brazil nut flour fermented for 4, 8, and 12 h, respectively.

NFBNF showed the highest inhibition of α‐amylase (27.06%) among Brazil nut samples. Fermentation exceeding 4 h significantly reduced (*p* < 0.05) the inhibitory activity. No inhibitory activity against α‐glucosidase was detected for fermented or non‐fermented Brazil nut samples (Table 3). This trend may be explained by the consumption of phenolic compounds, amino acids, and other molecules with inhibitory activity by the yeast, which utilizes these compounds as energy sources to support its growth and metabolism during fermentation.

Several studies demonstrated that phenolic compounds can inhibit α‐amylase and α‐glucosidase. The possible mechanisms involve competition with substrates or interactions between amino acid residues from the catalytic site of the enzyme and hydroxyl groups from phenolic compounds, forming hydrogen bonds and π–π interactions (de Paulo Farias et al. 2021; Miao et al. 2015). In the present study, babassu flour fermented for 4 h (FBMF‐4 h) showed a significant reduction in α‐amylase inhibition compared to the non‐fermented control (NFBMF), with values decreasing from 68.61% to 46.58%. This inhibitory activity further declined at 8 h (34.14%) and partially recovered at 12 h (65.11%), suggesting dynamic changes in enzyme‐interacting compounds during fermentation. Interestingly, α‐glucosidase inhibition in babassu flour increased at 8 h of fermentation (40.26%), exceeding the control (33.05%), and remained elevated at 12 h (35.87%), indicating that mid‐fermentation (8 h) may enhance the release or formation of bioactives targeting this enzyme specifically.

For Brazil nut flour, the fermentation consistently reduced α‐amylase inhibition from 27.06% in the non‐fermented sample (NFBNF) to 16.59% after 12 h (FBNF‐12 h). α‐Glucosidase inhibition, in turn, was not detected. These observations may be attributed to the differential availability of precursors for bioactive peptides and phenolics between the two matrices.

Small chain peptides are also related to the ability to inhibit digestive enzymes, forming electrostatic and hydrogen bonds with the enzymes and suppressing their activity. Some studies reported that the presence of amino acids with –OH groups (Ser, Thr, and Tyr) and basic amino acids (Lys and Arg) are related to the inhibition of α‐glucosidase (Mirzaee et al. 2023). de Matos et al. (2022) underscore that α‐amylase inhibition could be related to the interaction between the active site of the enzyme and hydrophobic amino acids in the peptide chain. Inhibition could also be a result of chelating amino acids, capable of changing the enzyme structure by removing calcium ions. Thus, the specific fermentation durations (8 h for babassu and 8–12 h for Brazil nut) appear to promote the synthesis or accumulation of compounds with distinct inhibitory mechanisms toward each enzyme.

Andrade and de Castro (2023) found that plant‐based beverages formulated with brown rice, sorghum, and lentils in different proportions and fermented with *S. boulardii*‐17 were able to inhibit up to 74.67% of the activity of α‐amylase. However, the inhibition of the α‐glucosidase activity was lower compared to this work, reaching a maximum value of 7.93%. The authors attributed the inhibitory activity to the presence of isoflavones and flavones and their transformations during fermentation, such as the formation of aglycones.

## 4 Techno‐Functional Properties

The WHC and OHC, CGC, and EC results for NFBMF and the fermented samples are displayed in Table 4. Fermentation did not lead to any significant alteration (*p* > 0.05) in WHC or OHC of FBMF‐12 h compared to NFBMF. In a similar way, fermentation did not show a significant effect (*p* > 0.05) on any of these two properties for Brazil nut samples; samples fermented 12 h exhibited results statistically similar to NFBNF (*p* > 0.05). However, for the two substrates, a decreasing trend in both WHC and OHC was observed during the initial stages of fermentation, followed by a subsequent increase, returning to levels equivalent to the non‐fermented flours after 12 h. This behavior may be attributed to structural modifications induced by microbial activity. In the early stages, enzymatic action—particularly from proteases and carbohydrases released by *S. boulardii*—may partially degrade protein and fiber structures responsible for lipid or water binding. This could reduce the exposure of hydrophobic/hydrophilic groups and disrupt the matrix structure, leading to lower oil or water retention. However, after 12 h of fermentation, the increase in OHC and WHC may be due to the formation of new biopolymeric structures. These structures may expose additional non‐polar regions or form a porous network that enhances oil entrapment, thereby recovering the functional property. Similar trends have been reported in other fermentation systems, where microbial growth alters techno‐functional behaviors over time. Aquafaba proteins fermented with *Lactobacillus plantarum* MA2 exhibited a decrease in OHC after 3 h of fermentation, followed by an increase in subsequent fermentation times. The authors highlight that these variations in OHC may be influenced by the fragmentation of protein structures during fermentation, leading to the exposure of apolar and charged side chains. Depending on the extent of disintegration and the nature of these exposed residues, interactions with lipids may either enhance or reduce oil binding capacity at different time points (Bekiroglu et al. 2023).

**TABLE 4:** Techno‐functional properties of babassu mesocarp flour and Brazil nut flours before and after fermentation with *Saccharomyces boulardii*‐17.

Sample	WHC (g g^−1^)	OHC (g g^−1^)	CGC (g mL^−1^)	EC (%)
**NFBMF**	2.50^a^ ± 0.07	1.47^a^ ± 0.07	0.06^a^	31.39^a^ ± 0.23
**FBMF‐4 h**	2.24^b^ ± 0.07	1.37^a^ ± 0.03	0.06^a^	40.60^a^ ± 3.93
**FBMF‐8 h**	2.27^ab^ ± 0.13	1.39^a^ ± 0.02	0.06^a^	47.56^a^ ± 9.14
**FBMF‐12 h**	2.33^ab^ ± 0.10	1.42^a^ ± 0.01	0.06^a^	36.77^a^ ± 3.86
**NFBNF**	2.97^ab^ ± 0.18	3.24^a^ ± 0.03	0.06^a^	66.49^b^ ± 0,70
**FBNF‐4 h**	2.61^b^ ± 0.20	2.51^b^ ± 0.04	0.06^a^	78.14^a^ ± 3.60
**FBNF‐8 h**	3.14^a^ ± 0.06	2.70^b^ ± 0.03	0.06^a^	74.32^a^ ± 2.21
**FBNF‐12 h**	3.19^a^ ± 0.07	2.99^a^ ± 0.20	0.06^a^	75.63^a^ ± 1.79

*Note*: Results presented as mean (*n* = 3) ± standard deviation. Different letters indicate significant differences (*p* < 0.05) between samples in the same column (for one type of substrate). NFBMF = non‐fermented babassu mesocarp flour; FBMF‐4 h, FBMF‐8 h, and FBMF‐12 h = babassu mesocarp flour fermented for 4, 8, and 12 h, respectively; NFBNF = non‐fermented Brazil nut flour; FBNF‐4 h, FBNF‐8 h, and FBNF‐12 h = Brazil nut flour fermented for 4, 8, and 12 h, respectively.

Abbreviations: CGC, critical gelling concentration; EC, emulsifying capacity; OHC, oil holding capacity; WHC, water holding capacity.

No significant effect (*p* > 0.05) on the gelling capacity of babassu or Brazil nut samples was observed after fermentation; the CMC found was 0.06 g mL^−1^ regardless of the sample. The gel made from babassu mesocarp flour was firm and homogeneous (as shown in Figure S1a). In contrast, the gel produced from Brazil nut flour was notably dry and brittle, as shown in Figure S1b.

The highest value for the EC was detected in FBMF‐8 h (47.56%), although no significant differences (*p* > 0.05) were found between NFBMF and fermented samples. However, fermentation significantly (*p* < 0.05) and positively affected the EC values of Brazil nut flour. The fermented samples were able to emulsify more than 74% of the oil added in the systems, against 66.49% of emulsification found in NFBNF (Table 4). Fermentation may modify the solubility and conformation of native proteins, exposing hydrophobic and charged residues that contribute to improved interfacial properties. The increase in EC for NFBNF suggests that moderate fermentation enhances the techno‐functional behavior of the protein fraction. A similar trend was observed by Bekiroglu et al. (2023), who reported that aquafaba proteins showed improved emulsifying properties at moderate fermentation times (around 3 h).

## 5 Scale‐Up of the Fermentation Process in a Benchtop Bioreactor

The duration of fermentation was set at 12 h for the process scale‐up, based on the results obtained in the assays conducted in Erlenmeyer flasks. The scale‐up studies assessed yeast growth, bioactive properties, and the techno‐functional properties of the flours, comparing them with the results obtained in the fermentation conducted in Erlenmeyer flasks for 12 h (FBMF‐12 h or FBNF‐12 h).

The microbial cell count did not show significant differences when fermentation conducted in Erlenmeyer flasks was compared to fermentation in the bioreactor (7.70 CFU mL^−1^ for FBMS‐12 h and 8.10 CFU mL^−1^ for FBNS‐12 h). The results obtained exhibited consistency in microbial growth, an indication that the microorganism may have reached its maximum growth capacity in the substrates evaluated.

**FIGURE 3:**
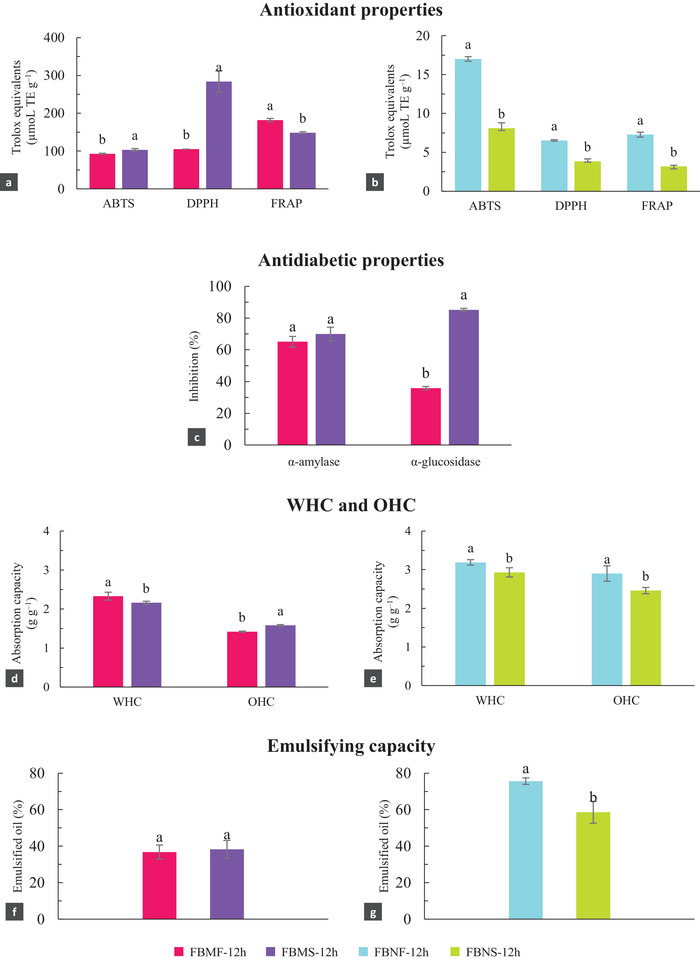
A comparison of the performance of babassu mesocarp flour and Brazil nut flour, each fermented for 12 hours in either an Erlenmeyer flask (FBMF‐12h and FBNF‐12h, respectively) or a benchtop bioreactor (FBMS‐12h and FBNS‐12h, respectively), for different assays: (a) Antioxidant properties (ABTS, DPPH, and FRAP) for FBMF‐12h and FBMS‐12h; (b) Antioxidant properties (ABTS, DPPH, and FRAP) for FBNF‐12h and FBNS‐12h; (c) Antidiabetic properties (inhibition of α‐amylase and α‐glucosidase) for FBMF‐12h and FBMS‐12h; (d) WHC and OHC for FBMF‐12h and FBMS‐12h; (e) WHC and OHC for FBNF‐12h and FBNS‐12h; (f) EC for FBMF‐12h and FBMS‐12h; (g) EC for FBNF‐12h and FBNS‐12h. Different letters indicate significant difference (*p* < 0.05) between samples in the same method (for one type of substrate).

Figure 3a,b presents the values obtained for the antioxidant potential of the fermented flours in the scale‐up process. For babassu flour, fermentation in the bioreactor had a greater impact on antioxidant activity than fermentation in Erlenmeyer flasks, resulting in higher values of µmol TE g^−1^ for ABTS and DPPH radical inhibition assays. The IC_50_ values of the flours fermented in the bioreactor were 1.47 mg mL^−1^ for the ABTS assay and 0.67 mg mL^−1^ for the DPPH assay. As for Brazil nut flour, the antioxidant activities were lower for the three assays evaluated. The IC_50_ values were 19.39 mg mL^−1^ (ABTS) and 16.33 mg mL^−1^ (DPPH) and were considerably higher than babassu flour, as expected.

The antidiabetic properties of babassu mesocarp flour were also positively affected by fermentation in the bioreactor. Regarding α‐glucosidase inhibition, it was only possible to determine the IC_50_ of the babassu flour (0.50 mg mL^−1^), as Brazil nut flour did not show inhibitory activity under any fermentation conditions. The same occurred with the α‐amylase assay, for which inhibition (%) values for Brazil nut flour did not reach 50%, whereas the IC_50_ of babassu flour was 3.72 mg mL^−1^ (Figure 3c).

WHC was slightly reduced in bioreactor fermentation, for both babassu and Brazil nut samples (Figure 3d,e). Minor modifications were observed in OHC, with increased values for babassu flour and lower values for Brazil nut flour. The scale‐up did not show any significant difference on the EC of the babassu mesocarp flour. However, it negatively affected the Brazil nut EC, which decreased from 75.63% to 58.58% (Figure 3g).

A reduction in the CMC value was also observed in Brazil nut flour, from 0.06 to 0.02 g mL^−1^. The CMC for babassu flour remained the same (0.06 g mL^−1^).

Although the microbial cell counts did not differ significantly between the fermentation scales, scale‐up can lead to changes in the functional and bioactive properties of fermented substrates due to physicochemical differences inherent to larger scale systems. Factors, such as oxygen gradients, mass transfer, heat dispersion, nutrient availability, and local microenvironments, may vary between Erlenmeyer flasks and bioreactors, even when overall microbial growth appears similar. These variations can influence the expression and activity of microbial enzymes, thereby affecting the release and transformation of phenolic compounds, proteins, and other metabolites responsible for antioxidant and antidiabetic activities, as well as techno‐functional properties. In the bioreactor, improved mixing and aeration may enhance the extraction or biotransformation of bioactive compounds, explaining the increased antioxidant and antidiabetic activities observed for babassu flour. Conversely, differences in oxygen availability, local pH, or substrate–enzyme interactions may account for the reduced WHC and EC, particularly for Brazil nut flour (Zhao et al. 2021; Hur et al. 2014). Therefore, the observed variations between scales are likely a result of the complex interplay between process conditions and microbial metabolism, which can modulate the release and transformation of functional metabolites independently of cell growth.

## 6 Conclusions

Babassu mesocarp and Brazil nut flours exhibited nutritional richness, bioactivity, and techno‐functional properties that highlight their potential as versatile ingredients in plant‐based formulations. Their ability to support the growth of *S. boulardii*‐17 during fermentation further reinforces their applicability in the development of functional, non‐dairy probiotic carrier products. Among the fermented samples, 12 h of fermentation yielded the most favorable results across both matrices. The extracts from fermented samples exhibited notable bioactivities, including antioxidant and antidiabetic effects, especially in babassu flour, whose bioactive potential was further enhanced under bioreactor conditions. In terms of techno‐functional properties, Brazil nut flour showed superior performance overall, while fermentation improved EC in both substrates. Babassu flour retained its gelling ability at low concentrations and showed increased protein content and amino acid diversity post‐fermentation. Although scale‐up fermentation enhanced the bioactivities of babassu flour, it led to a reduction in the same for Brazil nut flour. These findings highlight the potential of Amazonian plant‐based matrices as functional and bioactive food ingredients. In future studies, the fermented extracts will undergo in‐depth chemical characterization using chromatographic and mass spectrometry techniques to identify and quantify phenolic compounds, bioactive peptides, and other phytochemicals. This approach will enable a clearer understanding of the relationship between specific metabolites and the observed functional and health‐promoting properties. Additionally, the bioaccessibility of these compounds will be investigated through simulated gastrointestinal digestion to evaluate their stability and potential bioavailability under physiological conditions.

## Author Contributions

**Letícia Nunes da Cruz**: writing – original draft, visualization, investigation, formal analysis, data curation. **Beatriz Veltre Costa**: data curation, visualization, investigation, formal analysis. **Caroline Lopes**: investigation, data curation, formal analysis, visualization. **Cecília Midori Hosoda Henriques**: data curation, investigation, formal analysis, visualization. **Ruann Janser Soares de Castro**: writing – review and editing, supervision, resources, project administration, methodology, funding acquisition, conceptualization.

## Conflicts of Interest

The authors declare no conflicts of interest.

## Supporting information




**Supplementary Figure**: jfds70554‐sup‐0001‐figureS1.docx
