# Supplementary material for: Effects of Fermentation on the Bioactive, Functional, and Technological Characteristics of Babassu and Brazil Nut Flours
Source: J Food Sci. 2025 Sep 11;90(9):e70554. doi: 10.1111/1750-3841.70554 (PMC12424274; doi:10.1111/1750-3841.70554)
Supplement: Supplementary file 1 — Supplementary Figure: jfds70554‐sup‐0001‐figureS1.docx [file JFDS-90-0-s001.docx]

| 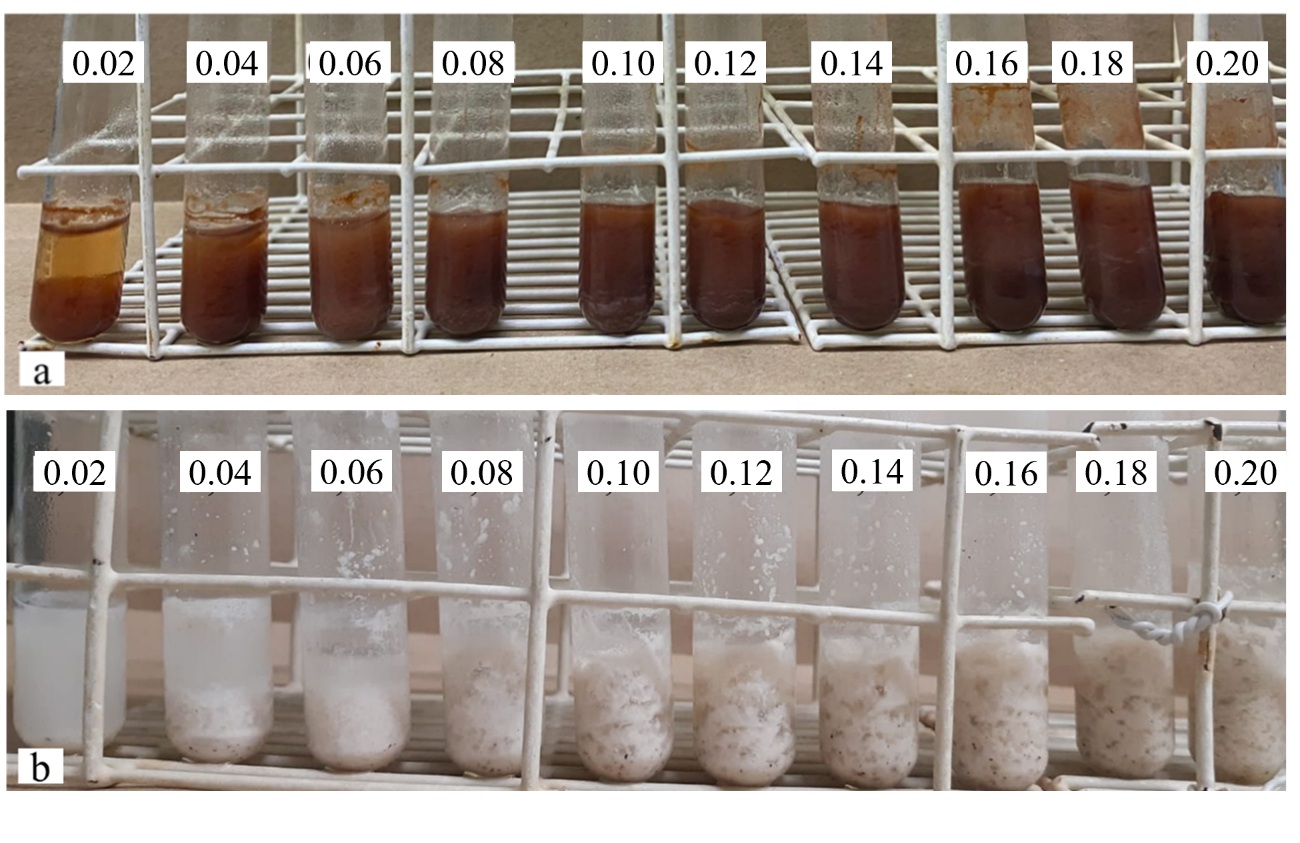 |
| --- |

**FIGURE S1** Gels formed from suspensions at different concentrations (0.02 – 0.2 g mL^– 1^) of babassu mesocarp flour (a) and Brazil nut flour (b). For all samples, regardless of fermentation time, the CGC was found at 0.06 g mL^–1^.
